# OpenAI’s Sora in intensive care medicine: opportunities and challenges

**DOI:** 10.1097/JS9.0000000000001809

**Published:** 2024-06-19

**Authors:** Nan Zhang, Qipeng Shao, Wanqing Li, Kunming Cheng, Haiyang Wu, Cheng Li

**Affiliations:** a Department of Gynecological Tumor Ward, The Third Affiliated Hospital of Zhengzhou University; b Department of Intensive Care Unit, The Second Affiliated Hospital of Zhengzhou University; c Department of Orthopaedics, The First Affiliated Hospital of Zhengzhou University, Zhengzhou; d Department of Orthopaedics, Ganzhou People’s Hospital, Ganzhou; e Department of Operating Room, Xiangyang Central Hospital, Affiliated Hospital of Hubei University of Arts and Science, Xiangyang; f Department of Clinical College of Neurology, Neurosurgery and Neurorehabilitation, Tianjin Medical University, Tianjin; g Department of Spine Surgery, Wangjing Hospital, China Academy of Chinese Medical Sciences, Beijing, China; h Center for Musculoskeletal Surgery (CMSC), Charité-Universitätsmedizin Berlin, Corporate Member of Freie Universität Berlin, Humboldt University of Berlin, Berlin Institute of Health, Berlin, Germany

*Dear Editor*,

Following the release of ChatGPT and DALL-E, OpenAI unveiled yet another groundbreaking technological advancement on 16 February 2024: Sora^1^. This artificial intelligence (AI) model possesses the remarkable ability to generate realistic or imaginative scene videos based on textual prompts. Sora’s text-to-video capabilities, characterized by their lifelike fidelity, immediately garnered widespread global attention upon release^2^. While not the pioneer in text-based video generation AI, Sora transcends its predecessors in several ways. In Table 1, we have summarized the currently existing mainstream video generation models and compared their parameters including length of generated video, frames per second (fps), as well as resolution. Compared with other models, Sora’s main advantages are as follows: (1) Sora overcomes time constraints and is able to produce 60-sec high-definition videos based on text input, achieving a breakthrough in video length and clarity; (2) Sora could simulate real-world physical laws. Among the 48 video demos generated by Sora updated on the OpenAI official website, Sora is able to deeply understand the details of the existence of objects in the physical world and generate characters with rich emotions; (3) Sora, as a multimodal large model, is capable of handling various types of data. In addition to text-to-video conversion, Sora could also accept input prompts in other formats, such as text, images, and videos, and generate high-quality video outputs; (4) By leveraging the re-captioning technology of the DALL-E model, Sora can generate descriptive captions that enhance textual accuracy and overall video quality, ensuring long-term coherence of characters and backgrounds within the video.

**Table 1:** Comparison of mainstream text-to-video AI models.

Models	Developer	Release time	Open source or not	Length of generated video	Frames per s (fps)	Resolution
Sora	OpenAI	February 2024	No	60s	No data	1920×1080
Pika 1.0	Pika	November 2023	Yes	3–7s	8–24 Fps	1280×720
Gen-2	Runway	March 2023	Yes	4–18s	24 Fps	768×448
Stable Video	Stability AI	February 2024	Yes	2–5s	3–30 Fps	576×1024
Emu Video	Meta	November 2023	No	4s	16 Fps	512×512

AI, artificial intelligence.

Foreseeably, such prowess harbored by Sora heralds unprecedented opportunities and challenges within the medical domain, notably in critical care medicine. Thus, this study undertakes a preliminary exploration of Sora’s prospective impacts in critical care medicine, encompassing diverse facets such as patient-physician communication, procedural training, rehabilitation guidance, remote healthcare, and the visualization and dissemination of scholarly endeavors.

## Patient-physician communication

Sora-generated videos serve as a conduit for imparting pertinent medical knowledge and caregiving skills to critically ill patients and their kin^3^. By elucidating the etiology, progression, and therapeutic modalities of ailments through video demonstrations, patients’ comprehension of their conditions is augmented, thereby fostering compliance with treatment regimens.

## Procedural training

Sora videos find utility in training healthcare practitioners, particularly in the realm of intensive care^4,5^. By showcasing real-life cases, surgical procedures, instrument handling, and nursing techniques, healthcare personnel attain a more intuitive grasp of diagnostic and therapeutic approaches to diseases, thereby enhancing their skill set and professional acumen.

## Rehabilitation guidance

Sora-generated videos serve as a tool for furnishing rehabilitation guidance to critically ill patients and their families. Through the exhibition of rehabilitation exercises and caregiving techniques, patients are better equipped to comprehend the rehabilitation process, thereby ameliorating its efficacy.

## Remote healthcare

In exigent circumstances such as when critically ill patients necessitate remote medical support, medical videos can be instrumental in facilitating teleconsultations, monitoring, and guidance. Through video technology, experts can remotely assess patients’ conditions and monitoring data, furnishing timely diagnostic advice and support to frontline healthcare personnel, thus elevating the standard of care and survival rates among critically ill patients.

## Visualization and dissemination of scholarly endeavors

Sora videos vividly showcase research findings, rendering the research process, experimental results, and conclusions in a visual format. Beyond traditional academic publishing and conference presentations, Sora videos can be disseminated widely through online platforms and social media channels, garnering increased attention and engagement in scholarly research endeavors.

Despite the manifold potential advantages, from the perspective of critical care physicians, Sora-generated videos entail certain challenges. Firstly, distinct from text generation models, video generation AI models necessitate a higher degree of sophistication, mandating an enhanced capacity for comprehending causal relationships, spatial intricacies, and temporal variations. Given the rapidly fluctuating nature of critically ill patients’ conditions, accurately simulating complex critical care scenarios constitutes the linchpin for Sora’s proliferation in critical care medicine. Secondly, akin to other generative AI tools, the process of data acquisition and utilization by Sora mandates stringent safeguards to uphold user privacy and data security, precluding the inadvertent disclosure of sensitive information to third parties or its misuse. This necessitates bolstering controls and oversight pertaining to data handling and user information protection. Moreover, within the domain of critical care medicine, patients’ conditions are inherently intricate, and the volume of diagnostic and monitoring data is substantial. Sora-generated videos may be susceptible to biases or contain misleading or erroneous information inherent in the model’s training data, potentially misleading critical care physicians’ treatment decisions and directly imperiling patients’ lives. Hence, enhancing the accuracy of AI-generated videos assumes paramount importance.

## Ethical approval

This study does not include any individual-level data and thus does not require any ethical approval.

## Source of funding

This study is supported by China Postdoctoral Science Foundation (2022M720385) and Beijing JST Research Funding (YGQ-202313).

## Author contribution

N.Z.: conceptualization, methodology, data curation, formal analysis, resources, investigation, writing—original draft; Q.S. and W.L.: conceptualization, methodology, data curation, formal analysis, resources, investigation; K.C.: conceptualization, methodology; H.W. and C.L.: methodology, data curation, formal analysis, resources, investigation, writing—review and editing.

## Conflicts of interest disclosure

The authors declare no conflicts of interest.

## Research registration unique identifying number (UIN)

1. Name of the registry:
2. Unique Identifying number or registration ID:
3. Hyperlink to your specific registration (must be publicly accessible and will be checked):

## Guarantor

Nan Zhang, Haiyang Wu, Cheng Li.

## Data availability statement

The data underlying this article will be shared by the corresponding author on reasonable request.

## References

[1] Cheng K Wu H Li C . ChatGPT/GPT-4: enabling a new era of surgical oncology. Int J Surg 2023;109:2549–2550. 37195797 10.1097/JS9.0000000000000451 PMC10442081

[2] O’Callaghan J . How OpenAI’s text-to-video tool Sora could change science - and society. Nature 2024;627:475–476. 38472485 10.1038/d41586-024-00661-0

[3] Waisberg E Ong J Masalkhi M . OpenAI’s Sora in ophthalmology: revolutionary generative AI in eye health. Eye (Lond) 2024. Published online April 30. 10.1038/s41433-024-03098-x PMC11383950 38693296

[4] Waisberg E Ong J Masalkhi M . Concerns with OpenAI’s Sora in medicine. Ann Biomed Eng 2024. Published online April 1. 10.1007/s10439-024-03505-0 38558354

[5] Waisberg E Ong J Masalkhi M . OpenAI’s Sora in medicine: revolutionary advances in generative artificial intelligence for healthcare. Ir J Med Sci 2024. Published online April 4. 10.1007/s11845-024-03680-y 38570404

